# What matters for cooperation? The importance of social relationship over cognition

**DOI:** 10.1038/s41598-020-68734-4

**Published:** 2020-07-16

**Authors:** Rachel Dale, Sarah Marshall-Pescini, Friederike Range

**Affiliations:** Domestication Lab, Konrad Lorenz Institute of Ethology, University of Veterinary Medicine, Veterinaerplatz 1, 1210 Vienna, Austria

**Keywords:** Social evolution, Psychology

## Abstract

Cooperation is vital for the survival of many species and has been extensively researched at the ultimate level however, there is a considerable degree of variation within a given species in the extent of cooperative behaviours exhibited. Possible factors that have been discussed to contribute to this variation are the social relationship between the cooperating individuals, but also non-social factors such as inhibitory control. Investigating the performance of wolves, a highly cooperative species, in three experimental cooperative tasks; a coordination (string-pulling) task, a prosocial task and an inequity aversion task, we found that the social relationship between the partners had the largest effects on all tasks, while non-social factors (inhibition, learning speed, causal understanding and persistence) had rather unpredicted, or no effects. The results support the potential importance of relational factors, rather than motivation and cognitive abilities, in driving cooperative interactions.

## Introduction

Cooperation is essential for humans and many non-human social species, playing an important role in individual fitness. While cooperation per se does not require complex cognition^1^, for example cooperation in bacteria and eusocial insects, authors argue that some types of cooperation in more socially complex species, particularly involving efficient coordination between partners and time delays between investment and compensation, brings added complexities that likely require certain cognitive abilities such as inhibitory control, associative learning and individual recognition^2^. However, although these cognitive pre-requisites have been theorised, there has been little experimental testing to date^2–4^.

Furthermore, some have debated that emotional bookkeeping may maintain stable cooperation without complex cognition^5^. This hypothesis proposes that when there are repeated social exchanges with specific partners, emotional states become associated with those partners, which allow long-term tracking (bookkeeping) of reciprocal exchanges. This concept is supported by research in humans which has found that repeated cooperative interactions develop a cooperative intuition, whereby their automatic first response is to be cooperative^6^, suggesting an emotional role in cooperation^7,8^. As such, social relationships may play an equally, if not bigger, role than cognition in cooperative decision making.

To achieve a deeper understanding of the influences on cooperative behaviour, we need to consider both social and non-social factors at the same time. In order to address this issue we gave wolves a battery of tests including three measures of cooperation, four measures of cognition, an experimental measure of social relationship (tolerance) and observational data on affiliation and dominance. The current study aimed to assess the relative proximate importance of social and cognitive influences on different aspects of cooperative interactions. The emotional states associated with specific partners are manifest in facets of the social relationship including affiliation, dominance and tolerance. These three facets constituted our social influences. Following Brosnan *et al*^2^ we refer to ‘cognition’ as an umbrella term covering perception and representation of the environment.

Cooperation is used as a general term referring to interactions that result in a net gain for all participants over time^9^. Specifically we identified three types of interactions that might be important to reach cooperation over time: (1) *coordination* in space and time in a task resulting in a benefit for both individuals (2) *prosocial* behaviour i.e. a voluntary action that benefits another, thought to be important in the initiation of cooperative interactions as well as cooperative acts involving a delay and reciprocity^10,11^ and finally (3) *inequity aversion* i.e. a negative reaction to an inequitable outcome, which ensures equitable sharing of the resources over time^12^.

Coordination, prosociality and inequity aversion (herein referred to as ‘cooperative interactions’) have now been demonstrated in a number of species in experimental settings (e.g.^13–16^).

Interestingly though, with a now rather large amalgamation of different studies in non-humans, it has emerged that even within species, there are large variations in the degree that these cooperative interactions are observed^17^. Therefore, an interesting avenue we are now at the forefront of, and that is little investigated to date, is to identify the key factors important for animal decision making in such cooperative tasks. Which social and/or cognitive factors lead certain individuals or dyads to be more coordinated, prosocial or inequity averse than others?

Amongst the potential influencing factors, the role played by the social relationship between partners has been increasingly considered in recent years. If cooperation and reciprocity are maintained via emotional bookkeeping^5^, we would predict dyads with stronger affiliative bonds, and therefore more positive affective associations between them, to be better at coordinating their actions, more prosocial, and less inequity averse. Indeed some studies have investigated the role of affiliation, a measure of social bond (coordination;^18–21^, prosociality;^22,23^, inequity aversion^24^;) and/or tolerance around a food resource (coordination^18,25,26^;) with many finding an effect.

Interestingly, the role of dominance rank on cooperation has been more extensively considered due to its large influence on social interactions in general. It has been suggested that subordinate individuals may be less willing to coordinate with^18^ or donate food to^27^ dominant individuals that always monopolise the gains, whereas dominant individuals would be predicted to be faster to react negatively to inequitable outcomes since they are unaccustomed to receiving the poor end of the deal^28^. The results have been somewhat contradictory regarding coordination (with many finding an effect^18,19,25,29^, but others not^20,21^), prosociality (where^22,27,30^ found an effect, but^31,32^ did not) and inequity aversion (with an effect in^28,33,34^ but not in^24^). These contradictory results may possibly be due to subordinate individuals using cooperation to invest in the relationship for future benefits or it may suggest the role of dominance is more species/methodology specific and requires further investigation.

Many of the aforementioned studies only considered one or two social relationship factors at a time, which may be misleading as the effects are often not all-or-none. The relative weights of each when included in the same analysis would provide a more complete picture. To our knowledge, only one study has investigated all three factors in the same analysis, probably due to practical challenges, especially in captivity, of testing non-affiliative pairs. Molesti & Majolo^18^ found that higher tolerance and a larger difference in rank, and to a lesser extent higher affiliation, between two individuals resulted in more successful cooperation in Barbary macaques. Interestingly, these findings suggest that tolerance and affiliation are not interchangeable, as is commonly assumed, and they may be having differential effects on coordination. Whether or not these findings are specific to this study and/or species remains to be investigated.

In addition to social relationships, non-social factors may affect performance on cooperative tasks. For example, inhibitory control, i.e. the ability to overcome the temptation of performing an immediately rewarding behaviour in favour of a delayed but more appropriate (and ultimately more rewarding) one, has been proposed to be one of the main factors affecting social decision making. In humans, as self-control allows inhibition of the impulse to act ‘selfishly’, individuals with higher levels of inhibition are more cooperative^35,36^. Similar mechanisms in social decision making may be at play in non-humans^37^ and indeed, in pet dogs those which took their time to respond on inhibition tasks were also more inequity averse than dogs that were faster (more impulsive)^38^.

However, with the exception of inhibitory control, until now little attention has been paid to other non-social factors which may influence cooperative interactions. Since most cooperative tasks involve aspects of apparatus learning and manipulation, causal understanding (defined as the ability to know the effects certain actions have on different objects), persistence, and learning speed may also potentially influence the performance of individuals in these tasks, since they may affect how animals interact with the apparatuses and their understanding of the task contingencies. Furthermore, teasing apart the influencing cognitive factors in controlled settings can identify the relevant factors to investigate in more challenging wild settings^4^.

In the current study, we set out to bring together results from multiple studies in the same population to investigate how far social and non-social factors influence the performance of wolves in a coordination task, a prosocial task and an inequity aversion task. Considering the scarcity of studies investigating the potential effects of different cognitive mechanisms on cooperative interactions, and the piecemeal results relating to the social effects, we adopted an integrated approach in which we tested multiple cognitive and social aspects on the cooperative tendencies of wolves. Wolves are an ideal model species to investigate aspects of cooperation since in the wild they rely extensively on cooperation for hunting, breeding and territory defence^39^. Furthermore, they show complex social relationships, with linear dominance hierarchies^40^, affiliative preferences and selective tolerance around food^41^. Additionally, experimental studies have revealed a certain level of sophistication in the cognitive domain^42,43^.

**Figure 1:**
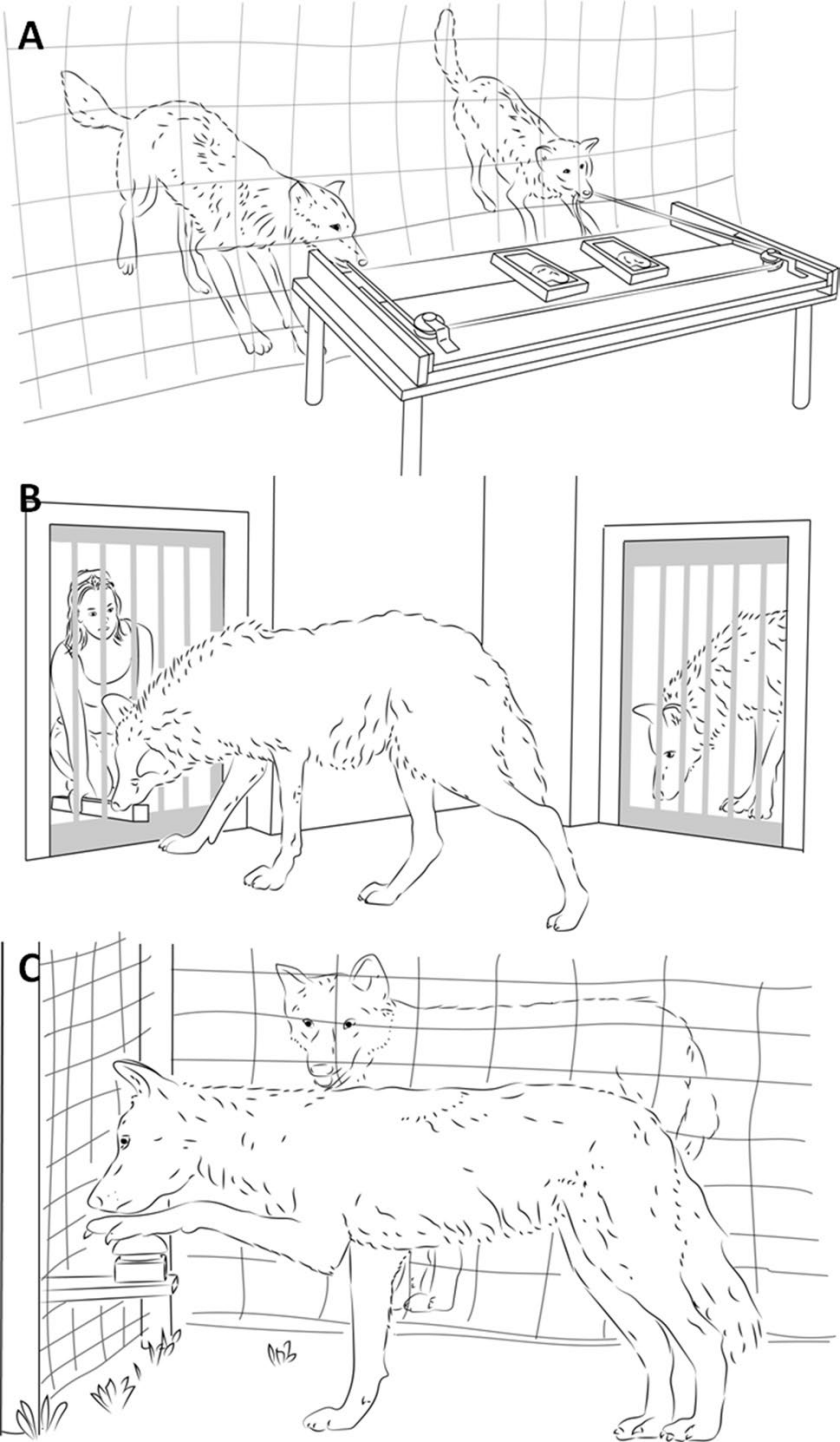
Images depicting the cooperative tasks. (**A**) the loose-string coordination task, where two individuals must pull simultaneously on either end of the rope to reach a reward, (**B**) the prosocial touch screen task, where after the subject (right) touches the giving symbol, the partner (left) is rewarded, and (**C**) the inequity aversion buzzer task, where the individuals alternately press a buzzer but while the partner is rewarded for the action, the subject is not. Image credit: Stephan Reber.

As mentioned above, we considered three aspects of cooperation: (a) coordination, (b) prosociality and (c) inequity aversion. Our measure of the animals’ capacity to coordinate with one another was their success in a variant of the commonly used loose-string paradigm (Fig. 1A), whereby two individuals are required to pull on either end of a rope in order to bring a baited tray situated on the other side of a fence within reach. If only one individual pulls on the rope, it becomes unthreaded and neither can acquire the reward. Success in a condition with two apparatuses, requiring coordination in both space and time, was the dependent measure. Prosociality was measured using a touch screen apparatus, whereby touching one symbol rewarded the partner’s enclosure (Fig. 1B) and the other symbol provided no reward. Finally the inequity aversion task required the animals to alternate in pressing a buzzer (Fig. 1C) with their paw to receive a reward, and the reward distribution was varied to be either equitable between the partners or not (see methods for more details).

The current sample of wolves have shown coordination^44^, prosociality^45^ and inequity aversion^28^ at the group level, but also demonstrated individual variation in these responses. The results from the previous studies revealed some effects of social relationship which explain part of the variation. Specifically, dyads with higher cooperative success were those closer in rank and higher in their affiliative bonds^44^. Additionally, subjects which were much more dominant to their partner were more inequity averse^28^. However, these previous studies only considered one or two factors at a time and did not consider tolerance or non-social factors at all.

Using social information on affiliative and dominance relationships (collected via focal observations of spontaneous interactions within the pack) as well as on tolerance around a food-source (collected experimentally) on the same wolves, we simultaneously modelled these multiple dyadic social influences on each of the cooperative interactions described above. Based on previous findings summarised above, we predicted that dyads with higher affiliation and tolerance to be more prosocial, better at coordinating their actions at the string-pulling table and less inequity averse. Furthermore, we expected rank to influence coordination and inequity aversion, with dyads closer in rank coordinating more successfully and more dominant individuals being more inequity averse. We predicted wolves dominant to their partner to be more prosocial than subordinates^27^, but expected dominance relationships to have less of an influence than affiliation and tolerance.

At the individual level, we also analysed the effects of measures of the aforementioned non-social factors (inhibition, learning speed, causal understanding and persistence) on coordination success as well as propensity for prosociality and inequity aversion. The wolves which completed the cooperation tasks were also given a battery of inhibition tests^46^, which have previously shown to measure inhibitory control in pet dogs^47^. We predicted that, as in humans and pet dogs, wolves with better inhibitory control would perform better at cooperating in the loose-string task, show more prosocial responses and be more averse to inequity.

Learning speed was measured by providing the animals with two different objects, one baited, the other non-baited. Learning speed was determined by the total number of trials that the animals took to reach a criterion of choosing the baited object in 9 out of 10 trials within one session. We predicted that animals with a higher learning speed are better at learning task contingencies, for example the association between their action and the outcome, and therefore better able to cooperate.

Causal understanding was assessed in a task whereby they had to choose a baited object in a two-choice task based on noise or shape causal cues^48^. Given that the coordination task in particular required apparatus manipulations, some understanding of how the objects work and the effect their actions have on them, i.e. understanding that the rope would come loose if only one animal pulls on it, is likely to be useful for successfully solving the task. As such, we expected that a higher level of causal understanding would allow the subjects to better understand/operate the apparatuses, thus allowing them to more successfully coordinate.

Finally, we considered the influence of persistence, which in this context we mean ‘task directed motivation’. Persistence is one of the most important correlates of general problem solving abilities^49^, probably by providing more opportunities to understand/solve the tasks. For example, in the string-pulling task, having the persistence to continue trying, even with inefficient partners, may ultimately result in more success. The current sample of wolves were tested on their levels of persistence by measuring for how long they would interact with a baited object that was impossible to gain food from^50^. Due to the link between persistence and problem solving, we predicted those animals with higher persistence levels to be better able to solve the string-pulling task. We also expected them to be more willing to continue working for a partner in the prosociality task. However, we expected persistent animals to be less inequity averse, as they may be willing to operate the buzzer for longer despite receiving an unequal outcome.

Bringing together both the social and non-social influences on social tasks in non-humans will be crucial to fully understanding the proximate processes underlying cooperation.

## Results

A dyads’ coordination success was considered the percentage of trials in which they were able to obtain rewards from both apparatuses in the loose-string task^44^. Prosociality was measured as the number of trials in which the subject donated food to their pack-member partner^45^. Each dyad was tested with only one individual as the donor to avoid potential reciprocity effects. Inequity aversion was considered as the number of trials the subject worked for in the reward inequity condition, where the partner received a reward for pressing the buzzer but the subject received nothing^28^. See the methods for details on these measures.

In order to tease apart the potential effects of the multiple factors described above, we ran two analyses:

### Social factors

To investigate the effect of social relationship, we analysed the data at the dyadic level (meaning that the unit of analysis and all the data referring to it was the dyad; affiliation score for each dyad, rank difference of the individuals in the dyad and feeding tolerance within the dyad). Three models were run, one for each cooperation task, with the measure of coordination success/prosociality/inequity aversion as described above as the dependent variable. Affiliation (rate of affiliative behaviours displayed during focal observations), rank distance (the difference in rank between the two individuals, as measured by the David’s score) and tolerance (percentage of a trial the dyad spent sharing a food resource) were included as factors. See methods for details.

### Non-social factors

To evaluate the non-social factors, the animals were analysed at the individual level. Since some subjects were tested with more than one partner in the string-pulling task, to evaluate their individual performance their average success across all partners was taken as the dependent measure. The subjects in the prosociality and inequity aversion tasks were tested with only one partner each. Inhibitory control was broken down into three different facets taken from a principal component analysis of results from the battery of tests (see^46^ and methods): motivation, flexibility and perseverance. Each of these facets was included as separate factors in the model. Furthermore, causal understanding (mean success at choosing the baited object based on causal cues)^48^, learning speed (average number of sessions to reach criteria on the object discrimination task) and persistence (amount of time spent interacting with a baited, but impossible to open, object)^50^ were also included in the model.

For each analysis, a Generalised Linear Mixed Model was run, followed by a model comparison approach based on AICc and a model averaging approach (MuMIn package in R) in order to evaluate the relative importance of each variable^51^ and to obtain the final statistics (Table 1). The results from the model comparisons can be found in the supplementary materials (Supplementary Tables S2, S3).

**Table 1:** Results from the analysis into the factors affecting dyadic coordination, prosociality and inequity aversion.

Experiment	Factor	RVI	Z-value	*p*-value
Coordination	**Affiliation**	**0.93**	**2.582**	**0.009**
	**Rank distance**	**1.0**	**4.124**	** < 0.0001**
	Tolerance	0.11	0.554	0.58
	Inhibition–motivation	0.04	0.314	0.754
	Inhibition–flexibility	0.03	0.110	0.913
	Inhibition–perseverance	0.08	0.569	0.569
	Causal understanding	0.05	0.572	0.567
	**Persistence**	**0.79**	**2.522**	**0.012**
	Learning speed	0.04	0.513	0.608
Prosociality	**Affiliation**	**1.0**	**3.851**	**0.0001**
	Rank distance	0.09	0.521	0.602
	Tolerance	0.18	1.110	0.267
	Inhibition–motivation	1.0	0.147	0.883
	Inhibition–perseverance	1.0	0.092	0.927
	Causal understanding	1.0	0.369	0.712
	Persistence	1.0	0.131	0.895
	Learning speed	1.0	0.046	0.963
Inequity aversion	Affiliation	0.36	1.883	0.06
	**Rank distance**	**1.0**	**5.218**	** < 0.0001**
	**Tolerance**	**0.94**	**2.705**	**0.007**
	**Inhibition–motivation**	**0.67**	**2.441**	**0.015**
	Inhibition–flexibility	0.06	0.892	0.372
	Inhibition–perseverance	0.04	0.064	0.949
	**Causal understanding**	**0.37**	**1.918**	**0.05**
	Persistence	0.03	0.403	0.687
	Learning speed	0.04	0.399	0.689

The relative variable importance (RVI, 0–1) of each factor, Z-values and p-values are derived from the model averaging (see also Tables S2–S3). Statistically significant results are marked in bold.

**Figure 2:**
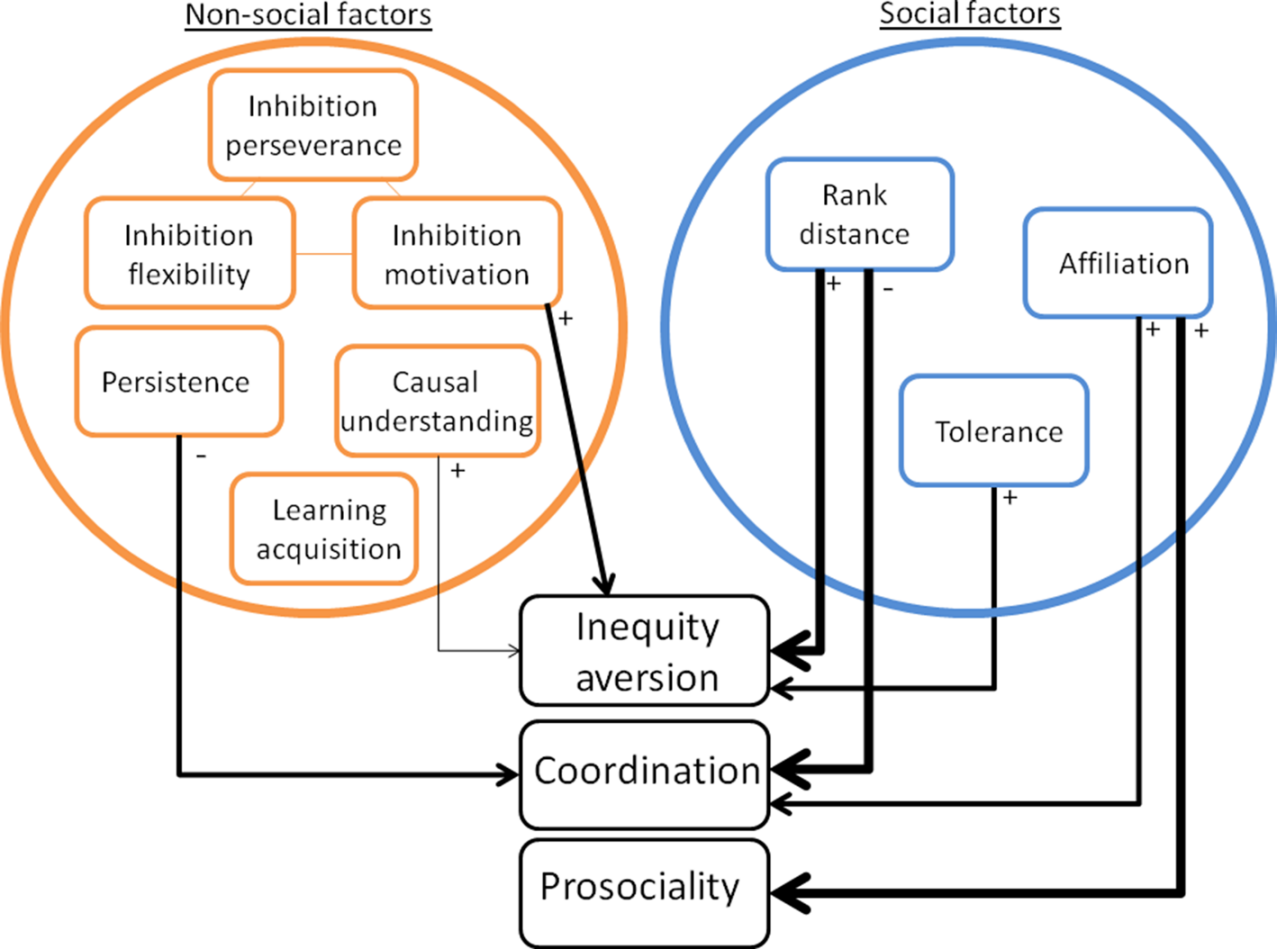
An infographic to illustrate the influences of both social and non-social factors on coordination, prosociality and inequity aversion. The thickness of the lines depicts the significance of the effect (*p* < 0.05, 0.01 or 0.0001), + /− symbols indicate the direction of the effect.

An overview of the results is depicted in Fig. 2.

The social relationship factors strongly influenced all three tasks (Table1, Supplementary Table S2). Dyads with higher affiliation scores (Supplementary Fig. S1) and lower rank distances (Supplementary Fig. S2) were more successful in the string-pulling task, but tolerance in a food-sharing task did not affect dyads’ performance. Individuals with higher affiliations scores with their partners were more prosocial towards them (Supplementary Fig. S4), but there was no effect of either rank or tolerance. Finally, the more dominant individuals were over their partners (Supplementary Fig. S5) and the greater tolerance shown to their partner in a feeding test (Supplementary Fig. S6) the greater their aversion to inequity.

Interestingly, considering the elements of object manipulation and learning required in all three tasks, the non-social factors had a surprisingly small effect (Table 1, Supplementary Table S3). Individuals which were less persistent attempting to solve an impossible task had higher average success in the string-pulling task (Supplementary Fig. S3), but no other non-social factors influenced coordination. Inequity aversion was influenced the most by non-social effects, with a moderately significant result of individuals with higher causal understanding showing higher inequity aversion and those with higher motivation in inhibition tasks also being more averse to inequitable outcomes (Supplementary Fig. S7). There were no significant effects of non-social factors on prosocial responses.

## Discussion

The striking message from our results is the overwhelming importance of the social relationship in the behaviour of the animals in all three cooperation-related tasks. These social factors showed far more significant effects than the non-social factors (Fig. 2) and in fact, three of the six non-social measures showed no effect at all on the dependent variables. By bringing together data from the same subjects on both social and non-social factors and analysing them in the same way for three different tasks, we have begun to tease apart the important influences on cooperative decision making in animals.

In support of the emotional bookkeeping hypothesis^5^, the affiliative bond a dyad shared strongly influenced their success on the coordination task and the extent of prosociality shown by one individual to their partner. Indeed, the fact that affiliation was the only factor to have an effect on prosocial responses in our sample is particularly interesting and highlights that this is the key metric for researchers to have in mind when deciding on prosociality study dyads, and/or interpreting results of such studies. Experimental cooperation should not be decontextualised; individual/dyadic history needs to be incorporated into the design and interpretation^8^. Overall, these findings suggest that positive associations with a partner, built up over repeated affiliative interactions, contribute to improved cooperation. These results corroborate those in humans which have also found social interactions to impact positively on cooperation and that emotions underpin this behaviour^6–8^. Furthermore, the current findings are in line with results from a range of other species (chimpanzees, macaques, kea, ravens) that suggest a clear role of social bond in successful cooperation^18,20,52,53^.

Interestingly, tolerance towards sharing a food resource only had an influence on inequity aversion, but not coordination or prosociality. Furthermore, affiliation did not have an effect on inequity responses. This finding is notable as it highlights that affiliative relationship and tolerance are not measuring the same dimension of the animals’ relationship (see also^18^). This is an important point as the two factors are regularly used interchangeably in the literature, with both being considered a measure of social bond. The results that affiliation, here measured as the amount of affiliative behaviours the individuals of a dyad show towards one another, is important for successful coordination and prosocial behaviour, suggest that this is a measure of social bond or ‘friendship’. On the other hand, dyads which showed more tolerance in food sharing were more inequity averse, suggesting that food tolerance measures are more related to attention towards food competition, rather than social bond. Although both relate to ‘social proximity’ and in fact, wolf dyads with higher affiliation scores do show more tolerance around food^41^, they are clearly not measuring the same construct, at least in some species.

The role of rank on cooperative behaviours has been the most studied of the social relationship factors, but has arguably the most inconclusive findings. Here we found that dyads closer together in rank were also more successful at coordinating, which is in line with studies on hyenas and chimpanzees^15,52^, but the opposite to results with macaques^18,25^. A clear interpretation of these findings remains elusive, however; upon inspection of our raw data there seems to be a combined effect of both rank and sex, with the male–female pairs that are close in rank coordinating the best. Our distribution of sex combinations did not allow us to include sex as a model variable, but it would be interesting to understand the interplay between rank and sex in larger samples.

While the relationship between rank and coordination requires further investigation, the significant effect of rank on inequity aversion was clearer, and as predicted. The more dominant animals were to their partner (i.e. the larger the rank distance between individuals in the dyad), the fewer trials they completed in the reward inequity condition, in which the partner received a reward but the subject did not. As previously suggested^28^, this may be because being treated unequally violates the expectations of the dominant individuals, resulting in a stronger aversion to the situation. Therefore inequity aversion in wolves, as in apes^33,34^, is dependent on the relative hierarchical position of the subject to their partner.

The social influences found in the current broad study expand prior studies and corroborate our previous findings, which only included one or two factors using different statistical techniques^28,44^ and are in line with the emotional bookkeeping hypotheses. Given that social relationships are important for each of the experimental cooperative tasks, it is therefore likely that they are also important for cooperation in natural conditions^4^. Indeed, given that our sample of captive wolves does not need to actively hunt or raise offspring, it can be predicted that the patterns found in this study would be even more explicit in wild populations.

In contrast, despite a number of papers theorising as to the specific cognitive requirements (e.g. inhibitory control and associative learning) for cooperation^2–4^, the experimental analysis of such factors had not been previously conducted. In the current study, these cognitive abilities had fewer and, where present, weaker effects on our tasks than the social factors. In fact, when comparing models including non-social factors, they were often no better than ‘null’ models (Supplementary Table S3). Nevertheless, there were still a few significant effects that are worthy of discussion.

Contrary to what we predicted, wolves with higher average success in the coordination task tended to be less persistent individuals. One possible explanation is that although persistence may be of benefit to initially learn the coordination task and to individual problem solving, in the two-apparatus condition the subjects already knew what action to do, but needed to show more sophisticated coordination. Therefore, reduced persistence may inadvertently allow subjects to hold back and work around their partner’s actions, thus resulting in better coordination. Alternatively, the raw data shows that of the six individuals with a success rate of above 65%, four of these had low persistence in the independent task and also needed additional string pulling training. . This suggests that individuals with low persistence might need additional training, which might in turn improve their coordination. So although training did not have a significant effect on coordination success in itself (see supplementary materials) and indeed some individuals coordinated well without training, it is possible there is an interaction between training and persistence, resulting in less persistent animals coordinating better.

It was further predicted that since all the tasks involved some form of apparatus manipulation and learning, a higher causal understanding and learning speed would help the animals in understanding the task contingencies, and therefore responding appropriately. Ultimately this was only the case for inequity aversion, whereby individuals which scored higher on causal understanding tasks tended to show more inequity aversion. This suggests that those individuals that better understood the task contingencies were more likely to be averse to the inequitable situation. It is not clear why this did not have an effect on prosociality and coordination. The attention load required to monitor the partners’ gains and actions, as well as the self, may be higher in the inequity aversion task, therefore making the link with causal understanding more evident.

We further found, as predicted, that inequity aversion responses were affected by inhibitory control. Those wolves with a higher motivation/activity level on the inhibition tasks were more inequity averse (i.e. they were faster to stop working when they were not rewarded but the partner was). The motivation component is a product of a principal component analysis on all of the measures from the four tasks which form the inhibition task battery^46^. This inhibition component is specifically formed of; (1) decision time in a reversal learning task, whereby the correct choice of two stimuli was reversed such that the previously correct response was now unrewarded, (2) decision time in a middle cup test, where the subjects could select two of three cups, two of which were rewarded, leading to one potentially unrewarded choice, (3) activity level in a buzzer task which involved moving away from a reward in a transparent box to press a buzzer which opened the box and 4) number of correct choices in the middle cup test. Therefore, it appears overall that individuals with faster decision times and higher activity levels were faster to demonstrate aversion to an inequitable situation. These findings are in line with that of a study on human participants which investigated a relationship between interactions in a prisoner’s dilemma game and individual variation in inhibitory control^54^. Accordingly, more impulsive people, as measured in a delay-discounting task, defect unequal offers more often than less impulsive people, who try to maintain offers for longer.

Unexpectedly however, the current findings are in contradiction to the only other non-human study to investigate a link between inhibition and inequity aversion. Brucks and colleagues^38^ found that pet dogs with higher motivation in these inhibition tasks actually showed less inequity aversion, that is; dogs that took more time to make decisions noticed the inequity earlier than those which made fast decisions. To understand these discordant findings, we compared the specific measures which loaded onto the motivation component of the wolf and pet dogs groups. The measures were almost the same with a few exceptions; for the dogs, but not the wolves, the latency to success on the box test loaded on the component while for the wolves the activity level on the buzzer task and the attention component from the middle cup loaded on the component. Despite some different loadings onto the motivation component, all measures involved an element of activity or motivation level suggesting that this does not explain the wolf-dog differences found here. An alternative possibility is that selection for specific traits during domestication is driving this disparity. Wolves are better at paying attention to and imitating conspecifics^55^ and have a better causal understanding^42^ than dogs. We also observe that wolves tend to be more active in tasks than the dogs are^44^. Therefore, we suggest that the wolves are simply much faster at understanding the task contingencies than the dogs and thus react more quickly to the inequitable situation. Overall, the wolf findings of the current task are more in support of the findings in humans^54^ that inhibitory control can positively impact success on cooperative tasks.

A limitation of our study is that we were unable to analyse the relationships between the cooperation tasks. A growing part of the literature has theorised on these relationships, with the hypothesis that to enable and maintain more complex forms of cooperation, mechanisms to start cooperation and to ensure equitable sharing of payoffs over time are required. Prosocial behaviour and inequity aversion have been identified as potentially crucial for this process in humans and non-humans alike^4,14,23,56,57^. Therefore, we acknowledge that assessing such connections is an important further step in establishing the bigger picture of cooperative processes. Unfortunately our sample size of dyads which had completed all tasks was too low for such an analysis; there were only five dyads which completed both the prosocial task and the coordination task, and only four dyads which completed the inequity aversion and coordination tasks. This will be an important question for future studies to address.

Furthermore, testing with one species in a particular context of course does not allow for the results to be generalised across the field. This situation has many advantages as it allowed us to run many tasks with the same individuals in a controlled manner. It is our hope that this kind of approach can be adopted by other facilities, and in other species, in order to collaboratively disentangle the influencing factors on cooperation, allowing for firmer conclusions across species.

In sum, wolves, with their dependence on cooperation, have provided an ideal model for assessing the relative importance of multiple social and non-social factors on success in three social tasks.

The measures of social relationship were all important explanatory variables in one or more of the tasks, corroborating suggestions from other species that the relationship between individuals can strongly impact on their cooperative behaviour. Whilst there were also some effects of the non-social factors, it was fewer than predicted and often in the direction contrary to what was expected. Overall, while not mutually exclusive in their influence, these findings lend stronger support to emotional^5,7^, rather than cognitive, mechanisms as key to promoting cooperation. Further research into these or similar factors in other species will be fruitful to fully understand social decision making in cooperative species.

## Materials and methods

### Subjects

The wolves (*Canis lupus*, N = 15; 10 M, 5F) used in the current studies (as described in^28,41,44–46,48,50^) were hand-raised in peer groups at the Wolf Science Center (WSC) in Ernstbrunn, Austria, after being separated from their mothers in their first ten days. They were bottle-fed and later hand-fed by humans and had continuous access to humans in the first five months of their life. After five months, they were introduced into the packs of adult animals and currently live in large 2000–8000 m^2^ enclosures. All animals receive training and/or partake in tests on a daily basis.

The animals’ care is in accordance with institutional and national guidelines. The research methods of each experiment were discussed and approved by the institutional ethics committee at the University of Veterinary Medicine, Vienna in accordance with GSP guidelines and national legislation (Cooperation: 01/04/97/2014, Prosociality: 10/08/97/2014, Inequity aversion: 10/09/97/2013, Social relationship measures: 03/01/97/2014, Inhibition: 10/12/97/2013, Persistence: 07/08/2016). The learning speed and causal understanding tasks were conducted prior to the requirement of an ethical statement but complied with relevant guidelines and regulations of the Tierversuchskommission am Bundesministerium für Wissenschaft und Forschung, the committee which allows research with animals in Austria.

#### Social cognition tasks

### Coordination

Coordination was assessed using the loose-string paradigm in outdoor testing areas; full methods can be seen in^44^. A 1.5 m × 75 cm table, with a 520 cm long rope passing through loops on the table, was placed on one side of a fence. 120 cm of each end of the rope lay on the other side of the fence, within the animals’ testing enclosure. If only one end of the rope was pulled, the rope slid out of the loop system and the other side of the rope became unavailable. On the table, 20 cm apart from each other were two food delivery areas, each containing one chunk of meat and one dead chick. In order to obtain this food reward, both subjects needed to each pull one end of the rope simultaneously to bring the food within reach (Fig. 1A).

Although the subjects took part in multiple conditions^44^, the current study used data from the two-apparatus condition. In order to progress to this condition, each dyad had to be previously successful in solving one apparatus on at least four of six trials in two sessions, therefore the two apparatus condition was the condition where the dyads had the most similar amount of experience and at least some knowledge of the task. It is also the condition in which, due to the need to coordinate in the choice of which apparatus to approach first, a greater degree of coordination between subjects is required. For these reasons, we considered it the most appropriate condition to use to tease apart which social and non-social factors may be important to the animals’ success. Twelve wolf dyads reached the necessary criteria to proceed to this condition (Table 2).

**Table 2:** Dyads and subject within the dyad (where relevant) which participated in each of the coordination, prosociality and inequity aversion tasks, as well as the social relationship metrics (affiliation, rank distance and tolerance) of each of those dyads.

Dyad	% Cooperative success	Number of prosocial trials		Number of trials during reward inequity		Tolerance—% time spent co-feeding	Affiliation score	Rank distance
		Subject	Score	Subject	Score			
Geronimo-Yukon	87.5	Yukon	7	Geronimo	8	38.72	0.52	3
Kaspar-Shima	54.76	Kaspar	1	NA	NA	54.17	0.752411576	20
Aragorn-Shima	40.28	Shima	6	NA	NA	22.42	0.523636364	14
Aragorn-Tala	88.89	Aragorn	44	NA	NA	57.06	1.817518248	3.2
Chitto-Shima	95.83	NA	NA	Chitto/Shima	8/12	69.96	1.229681979	5
Kaspar-Aragorn	20.83	NA	NA	Kaspar/Aragorn	1/30	28.61	1.089041096	5.9
Nanuk-Una	83.33	NA	NA	Nanuk	20	13.18	0.730769231	2
Kenai-Amarok	NA	Kenai	1	Kenai/Amarok	21/3	23.71	1.235294	− 2
Wamblee-Yukon	NA	Wamblee	24	Wamblee	1	33.52	0.91	− 3
Kaspar-Chitto	100	NA	NA	NA	NA	9.27	0.58	15
Kaspar-Tala	64.58	NA	NA	NA	NA	40.43	1.374193548	9
Tala-Shima	58.33	NA	NA	NA	NA	61.6	0.409556314	10
Tala-Chitto	100	Tala	21	NA	NA	55.46	2.0	5.9
Aragorn-Chitto	87.5	NA	NA	NA	NA	52.9	1.090909091	9
Ela-Etu	NA	Ela	6	NA	NA	66.75	2.025478	− 0.8
Etu-Maikan	NA	Etu	3	NA	NA	57.76	0.90566	− 3.8

In some dyads, both individuals were the subject of the inequity study; in these cases the subjects and scores are separated by a slash respectively. Negative values in the rank column indicates that the subject is of lower rank than the partner.

In the two-apparatus condition, two identical apparatuses were placed 10 m apart. The animals started each trial in compartments on the opposite side of the testing enclosure from the apparatuses. Before each trial, the experimenter called the animals’ attention and clearly placed the food on both apparatuses and then moved behind a screen out of sight. The animals were then released simultaneously into the testing enclosure and given a maximum of two minutes to solve the task. A total of six sessions of six trials each were conducted per dyad. A trial was considered a success if the dyad obtained food from *both* apparatuses within a trial. The score used in the current analyses was the percentage of successful trials in the two-apparatus condition.

Each individual had a different level of prior success on the task, given that they could differ in relation to the number of partners and conditions they had participated in. Therefore in order to control for this, the percentage of each individual’s success in all previous conditions with all prior partners was calculated. To take into account the prior success of both partners in the dyad, the mean previous success of the dyad was calculated as the previous success of individual A + previous success of individual B divided by 2. A linear mixed model was then run (using the lmer package in R) with percentage success on the two apparatus condition for each dyad as the response variable and mean percentage of previous success for that dyad as the factor. The model revealed no significant effect of previous success with the loose-string apparatus on success with the current dyad in the two-apparatus condition (χ^2^(1) = 0.237, *p* = 0.63) and was therefore not included as a factor in subsequent analyses.

### Prosociality

The prosociality task^45^ was conducted using a mechanical touch screen which simultaneously presented two visual symbols to the animals, one of which could be selected by the subject by pressing their nose against it. Each subject was randomly assigned two stimuli symbols; one of which delivered a reward to the adjacent ‘partner’ compartment (‘giving’) and one delivered no reward (‘control’). All subjects were already familiar with making discriminations on the touch screen, but were naïve to the posed question and symbols.

Nine subjects (Table 2) were first trained to choose the giving symbol by giving access to the partner room during this phase. Thus, when the subject selected the giving symbol they could then move into the partner compartment and receive the reward that was slid into the room by the experimenter. When the control symbol was chosen, no reward was given but 6–8 s of a white screen were presented and then the next trial started. Once subjects chose the giving symbol on 17/20 trials within one session, they moved on to the testing.

The testing procedure consisted of test (where the partner received the reward) and control conditions (where the partner was present but in a different compartment and therefore unable to receive the rewards) and each subject was tested in both conditions once with a pack member and once with a non-pack member^45^. In the current study, in order to assess the influence of social relationship measures on prosocial responses, only the test condition with the pack-member partner was used (i.e. where a pack-member was present in the partner room and could receive any rewards delivered by the subject, Fig. 1B). During the test condition, subjects were given 15 s per trial to choose a symbol and the session ended when the subject twice refused to choose a symbol within this time limit or after a maximum of 80 trials. The number of giving trials performed was taken as the measure of prosociality.

### Inequity aversion

Inequity aversion was assessed using a buzzer task^28^ where all subjects (N = 9) were first trained to press a buzzer with their paw (Fig. 1C) after a request by an experimenter pointing at the buzzer and saying ‘press’. Once subjects pressed the buzzer after the ‘press’ command 10 times in a row, they proceeded to testing.

During the test, individuals were placed in two adjacent outdoor enclosures. The buzzers could be moved in and out of these enclosures from the outside and so at the start of each trial, a hidden helper pushed the buzzer into the animal’s enclosure. The experimenter, standing in the middle and visible to the animals, then gave the press command up to 10 times, calling the animal’s name after the fifth command. If the animal pressed the buzzer after a command, the buzzer was retracted and the experimenter delivered the required food reward (depending on the condition). If the animal did not press the buzzer after all 10 commands, the session ended. The trials alternated between subject and partner, with a session always beginning with the partner and lasting for a maximum of 30 trials per animal, or until one of the animals refused to participate further.

Subjects participated in multiple conditions to control for effects of food quality, frustration, visibility and movement of the food^28^. The primary condition to measure inequity responses was the reward inequity condition, where the subject received no reward for pressing the buzzer and the partner received a high-value reward. The fewer the number of trials the subject is willing to press the buzzer in this condition, the stronger their inequity response. The number of trials performed by the subject in this condition was used in the current analyses (Table 2).

#### Social relationship measures

Regular focal observations of social interactions are conducted daily at the WSC when the animals are undisturbed in their home enclosures. Ten-minute focal samplings using the “all occurrences” method are carried out for all individuals, evenly distributed across days and times, using the Pocket Observer (3.2 Software) and are then imported into the Observer XT 10.5 program (both from Noldus Information Technology, Wageningen, The Netherlands).

Descriptions of all behaviours used for the affiliation and rank measures can be found in the ethogram in^41^. The data from these observations is analysed once per year as this provides sufficient data to calculate the social relationship scores. All social relationship data was taken from the year in which the cooperation task was conducted.

### Affiliation

An ‘affiliation score’ was calculated for each dyad^58^, representing the bidirectional frequency of affiliative behaviours exchanged within a dyad, divided by the observation time (hours) for that dyad.

$$ {\text{Affiliation}} = \frac{{{\text{A}}_{{\text{i}}} + {\text{B}}_{{\text{i}}} }}{{{\text{A}}_{{\text{j}}} + {\text{B}}_{{\text{j}}} }} $$

where i represents the frequency of affiliative behaviours and j represents the total number of observation hours.

### Rank distance

In order to characterise the dyadic dominance relationships between the two individuals, we calculated the David’s score^59^ for each individual and subtracted individual A’s score from individual B’s score to obtain the difference in rank for each pair. The David’s score has the advantage of accounting for the relative dominance positions of other individuals in the group when calculating the relative score of each animal and thus provides a good measure of the difference in rank of a dyad in the context of their group.

### Tolerance

Tolerance tests were conducted as part of a larger investigation into food sharing in wolves^41^. In this simple test two individuals were placed in separate, but adjacent side enclosures of the outdoor testing area. An experimenter placed a bowl (40 cm diameter) baited with chunks of meat and dry dog food in a central enclosure, equidistant from the doors of the two side enclosures. The experimenter then left the enclosure and the animals were simultaneously released into the central enclosure via a sliding door system. Two trials were conducted per dyad and each trial lasted for 2 min, or until the food was finished. The mean percentage of both trials the animals spent peacefully sharing the food (i.e. feeding together from the bowl without signs of aggression) was taken as the measure of tolerance and used in the current analyses.

### Non-social factors

#### Inhibition

The test battery to measure inhibition was composed of four separate tasks, each aimed at assessing different facets of inhibitory control^46^.

- Buzzer test
  In this task the animals first learnt to press a buzzer (a behaviour already familiar from the inequity task) in order to open an opaque 25 cm^3^ box which contained a reward. When the subject could press the buzzer without help from the experimenter in seven consecutive trials, they moved onto the test. In the testing phase the box was then made transparent and the buzzer was moved further from the box (2 m compared with 50 cm in training). Therefore, the individuals needed to refrain from manipulating the box with the visible reward, but move away from this to press the buzzer. At the start of a trial the subject was held by the collar 2 m away from the box while a helper baited it with a food reward. The subject was then released and able to press the buzzer to open the box. If they did not press the buzzer within 60 s, the trial ended and the experimenter called the animal back to the starting position. Five test trials were run per subject and the duration spent close to the box, latency to press the buzzer, duration of box manipulation and number of successful trials were coded.
- Box test
  A rectangular Plexiglas box (55 × 45.1 × 46.6 cm) with one open side was used in this task. A food reward was place inside the transparent box and then the animals were released 2 m from the box and were free to access the reward. After six training trials with visible baiting and an opaque box, six test trials were run where the box was now transparent and baiting of the box was not visible to the animals. The reward was either placed centrally in the box or ‘deep’ in the box, touching the wall opposite to the open side and the animals had 30 s to access the reward. Duration spent close to the box, latency to access the reward, number of successful trials and frequency of touches to the box were recorded.
- Middle cup test
  In this test, subjects could choose two of three transparent cups, of which two were baited. At the start of a session two warm-up trials were conducted, where only one cup was baited and when the animal was released from 2 m away they could knock the baited cup over and gain the reward. In the following test phase two cups were baited; either adjacent to one another or non-adjacent (i.e. the middle cup was empty). Now when the animal was released they were allowed to make two choices, after which the board containing the cups was pulled back behind a screen by the experimenter. Twenty trials were conducted with 10 for each condition (adjacent vs. non-adjacent) presented in a semi-randomised order. Frequency of correct choices, latency to make both choices and time spent in proximity to the cups were coded for analysis.
- Reversal learning test
  In this test subjects initially learned to discriminate between two objects, one which was rewarded and one which was not. In this acquisition phase the objects were pushed out from behind a barrier by an experimenter, whilst the subject was held 2 m away by a trainer. The subject was then released and allowed to make a choice by touching the object with their nose. After a correct choice the experimenter lifted the object and the subject could take the reward underneath. After an incorrect choice the subject was shown that the chosen object was not baited then the rewarded object was quickly lifted so that the subject could see the reward but could not take it. Criterion was reached when the subject chose the correct object on 9/12 trials within one session. After reaching criterion, the object contingencies were reversed such that the previously rewarded object was now unrewarded and vice versa. The ratio between correct choices in the last acquisition phase and the reversal phase was the primary measure in this test, but frequency of correct choices, latency to make a choice and duration spent close to the objects before a choice were also coded.
- Components of inhibitory control
  Since the inhibition battery produced multiple measures from multiple tests, all potentially informing on different facets of inhibitory control, Brucks et al.^46^, following the procedure of^47^, transformed the measures into z-scores and ran principal component analyses (PCA, see REF for details) first on each individual test using all measures from that test, and then an overall PCA including the components derived from the PCAs on the single tests. The overall PCA revealed three underlying components, which explained 71.7% of the variation in the data:
  - Motivation: choice time from the reversal learning test, decision time and attention in the middle cup test, activity level in the buzzer test. The component captures an animal’s attention, activity and speed in making a choice.
  - Flexibility: flexibility in behaviour in the box and reversal learning tests, persistence in the buzzer test. The component measures adaptability to change.
  - Perseveration: perseverance in the box test, persistence in the buzzer test. The component shows persistence with a selected behaviour.

The PCA gave each individual a score on each of these components. These three scores were used in the current analyses as measures of inhibitory control.

##### Causal understanding

In the causal understanding experiment^48^ subjects were tested on their understanding of both physical causal cues and human social cues. Only the results from the physical causal cues were used in the current study. Subjects were tested in the outdoor testing area, with a testing table placed against the fence on the other side from the animals. A concealer object or container (depending on the condition) was placed at either end of the table, one of which was baited and the other was empty. The procedure was a basic choice task, where after receiving a cue, the subject could choose one of these two objects.

At the start of each session pre-trials were run where a piece of sausage was placed on the left or right side of the table and the animal was allowed to choose a side. If they chose correctly four consecutive times, they were deemed motivated and attentive enough to proceed to the experimental trials. In the experimental trials one of the objects was baited out of view of the subject, behind a lowered screen and placed on either the left or right, with the empty object on the other side. The screen was then lifted, a cue was presented and the table was pushed forwards such that two targets on the left and right sides of the table were pushed through to the animal’s side of the fence. The subject could then indicate their choice by touching the corresponding target with their nose. The causal cues were a) *noise* where each container was shaken one at a time, one produced a noise indicating it contained a reward but the other remained silent and b) *shape* where two wooden shapes were presented, one of which was inclined indicating a reward underneath while the other lay flat against the table. Subjects received two trials of each condition across two testing sessions and their percentage success across both trials was used as the measure of causal understanding in the current study.

##### Persistence

To test persistence a large perforated “lion feeder” ball was baited with strong-smelling sausage and meat and anchored to the ground in the home enclosure of the subject, with all other pack-members moved to a different enclosure for the period of the session^50^. The reward within the ball was inaccessible to the animals. To ensure consistent and high food motivation, all subjects were tested in the morning and were not fed the night before. After the object was placed in the enclosure, the subject was released back and free to interact with the apparatus. The test stopped after the animal had stopped interacting with the ball for more than five minutes. The duration (in seconds) the animals spent manipulating the ball (physically manipulating the object with paws, snout, mouth or any combination of these three, excluding just sniffing^50^) was considered the measure of persistence and used for the current analyses.

##### Learning speed

Learning speed was assessed using a standard discrimination learning procedure. This procedure was similar to that of the reversal learning task described above, except that there was no reversal component.

Subjects learned to discriminate between two objects, one which was rewarded and one which was not. At the start of each trial the objects were placed on the ground 2 m apart by an experimenter whilst the subject was held 3 m away by a trainer, who had their eyes closed. The subject was released once the experimenter had retreated to stand 1 m in front of the objects with the back turned towards them (i.e. she could not see the wolf) and allowed to make a choice by touching the object with their nose or turning the object over. After a correct choice the experimenter lifted the object and the subject could take the reward. After an incorrect choice the baited object was quickly removed by the experimenter and the empty container was shown to the subject. The subject was then called back by the trainer and then next trial was begun. Each session consisted of 10 trials and sessions continued until the subject chose the baited object on 9/10 trials in two consecutive sessions.

The number of trials each individual took to reach criterion was taken as the measure of learning speed in the current analysis.

The individual scores from each of these tasks can be seen in Table 3.

**Table 3:** Individual scores for each of the non-social factors and the averaged individual scores on the coordination task.

Individual	Mean % cooperative success	Mean % causal success	Inhibition–motivation	Inhibition–flexibility	Inhibition–perseverance	Persistence (s)	Learning speed (#trials)
Kaspar	46.2962963	50	*− *0.82447	*− *0.13251	*− *0.28097	722.4	34
Aragorn	25	50	0.099015	0.058981	0.727709	940.8	36
Shima	42.66666667	75	*− *0.5354	*− *0.65493	0.201336	443.2	22
Tala	67.30769231	87.5	*− *0.90858	*− *0.16861	*− *0.66701	169.2	26
Chitto	95.13888889	87.5	*− *0.06543	*− *0.30992	0.78867	481.6	34
Nanuk	77.77777778	75	0.026743	0.441975	*− *0.25119	136.4	57
Una	77.77777778	37.5	*− *0.20011	0.670114	*− *0.70484	5.6	26.4
Geronimo	86.11111111	50	*− *0.89199	0.214973	*− *0.68609	58.4	54
Yukon	86.11111111	62.5	*− *0.07528	*− *1.21471	0.409275	78	26

##### Statistical analyses

All social measures were in reference to the dyad (e.g. the affiliation score of the dyad, the difference in rank between the two individuals of the dyad), where the non-social factors were scored individually. Therefore, to assess the effects of these categories we ran two separate analyses. Both analyses followed the same procedure.

In both cases, a Generalised Linear Mixed Model was run, followed by a model comparison approach based on AICc using the dredge function of the MuMIn package in R. This function generates every possible model, with all possible fixed effect combinations (see supplementary materials for details on controlling for collinearity). The output then provides each model with an AICc score and a weight, which can be used to select the best model. To evaluate the importance of each factor in explaining our dependant variable, we report the Z- and p- values based on model averaging (model.avg function in the MuMIn package) and additionally report the Relative Variable Importance (RVI) of each factor^51,60^. The RVI provides each variable with a value between 0 and 1 to denote its’ relative importance in the model (1 = highest importance). This approach was selected since it was possible the all factors may have influenced the response variables^60^.

## Supplementary information

Supplementary file1 (DOCX 4578 kb)

## Data Availability

Raw data is provided in the supplementary materials.
