# Supplementary material for: What matters for cooperation? The importance of social relationship over cognition
Source: Sci Rep. 2020 Jul 16;10:11778. doi: 10.1038/s41598-020-68734-4 (PMC7366628; doi:10.1038/s41598-020-68734-4)
Supplement: Supplementary file 1 — Supplementary file1 (DOCX 4578 kb) [file 41598_2020_68734_MOESM1_ESM.docx]

Title

What matters for cooperation? The importance of social relationship over cognition.

**Authors**

Rachel Dale^1^*, Sarah Marshall-Pescini^1^, Friederike Range^1^

**Supplementary materials**

Supplementary analyses

*Correlations*

Since an issue of collinearity was possible between the factors included in the models, we checked whether there were correlations between any of the variables using the *cor* function in R (Pearson’s method), and then assessing the matrix correlation using the Bartlett test. The social factors did not show any high correlations (Affiliation-Rank r= -0.39322367, Affiliation-Tolerance r= 0.43305973, Rank-Tolerance r= -0.09902595) and a Bartlett test on the correlation matrix was non-significant (χ²=3.51(3), p=0.32), suggesting the variables were sufficiently different for all variables to be included within the same model. Likewise, the non-social factors were not correlated with each other (Table S1) nor was the Bartlett test significant (χ²=16.89(15), p=0.33).

Therefore, for both the social and non-social analyses, all factors were included in the models.

Table S1. Correlation values between each of the non-social factors.

|  | Causal understanding | Inhibition-motivation | Inhibition-flexibility | Inhibition-perseverance | Persistence | Learning speed |
| --- | --- | --- | --- | --- | --- | --- |
| Causal understanding |  | -0.013239635 | -0.381258853 | 0.2710829 | -0.04932781 | -0.08829767 |
| Inhibition-motivation |  |  | 0.003201467 | 0.6465125 | 0.14433186 | 0.05563707 |
| Inhibition- flexibility |  |  |  | -0.5347447 | -0.10450044 | 0.51714926 |
| Inhibition- perseverance |  |  |  |  | 0.59826295 | -0.20867260 |
| Persistence |  |  |  |  |  | -0.13104875 |

*String-pulling training*

In the coordination task, those individuals which failed at the initial spontaneous condition with only one apparatus were given additional training prior to receiving the one apparatus condition again. Only once they were successful with one apparatus did they participate in the two-apparatus condition used in the current analyses. In order to assess whether this additional training had an effect on success in the later two-apparatus task, we ran a linear mixed model with mean cooperative success as the dependent variable and training (binomial yes/no) as the fixed effect. Whether or not an individual received prior string-pulling training did not impact their average success across partners on the two-apparatus condition (t(7)=1.933, p>0.05) and therefore was not included in further analyses.

Supplementary figures

**Coordination**


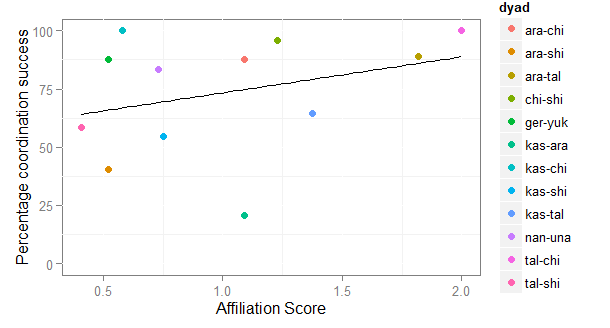


Fig. S1. The higher the affiliation score, the more successful a dyad was on the coordination task.


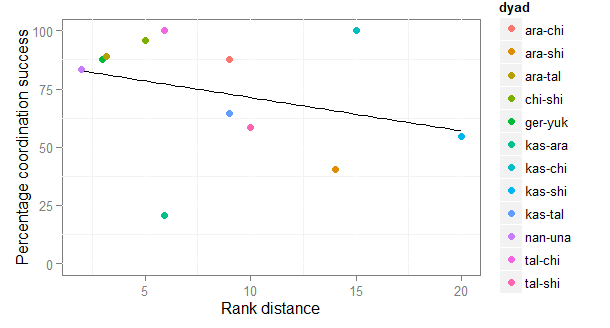


Fig. S2. The closer in rank a dyad was, the more successful they were on the coordination task.


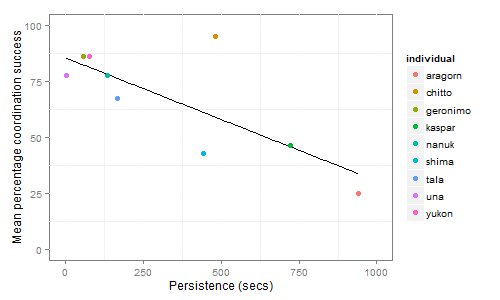


Fig. S3. The lower the persistence level of an individual, the more successful they were on the coordination task.

**Prosociality**


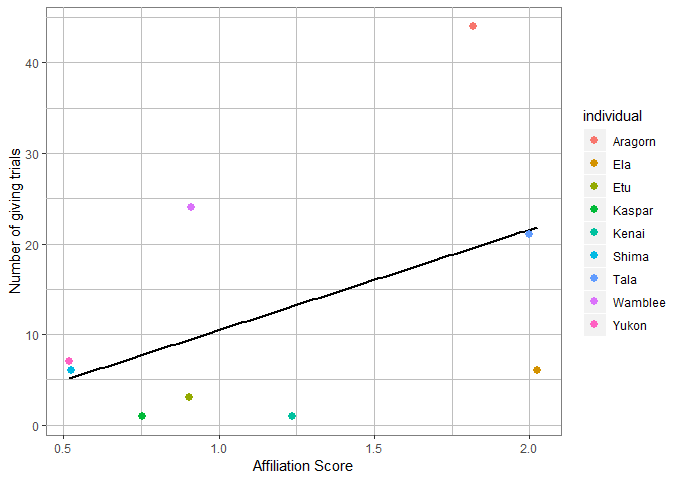


Fig. S4. The higher the affiliation score with their partner, the more prosocial the subjects were.

**Inequity aversion**

**
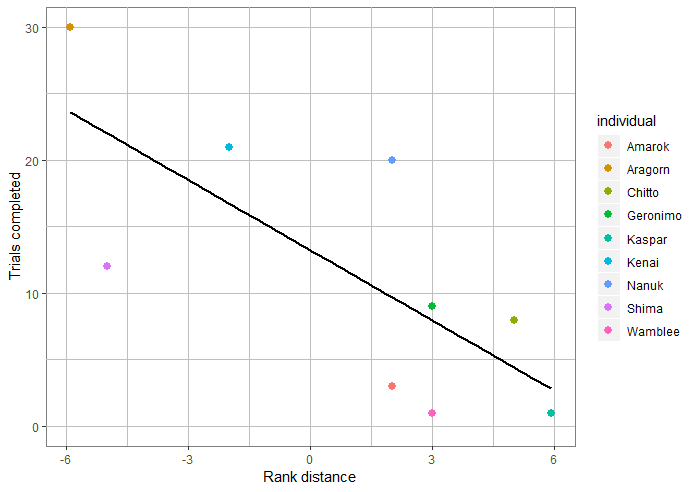
**

Fig. S5. The more dominant a subject was to their partner, the more averse they were to inequitable outcomes.


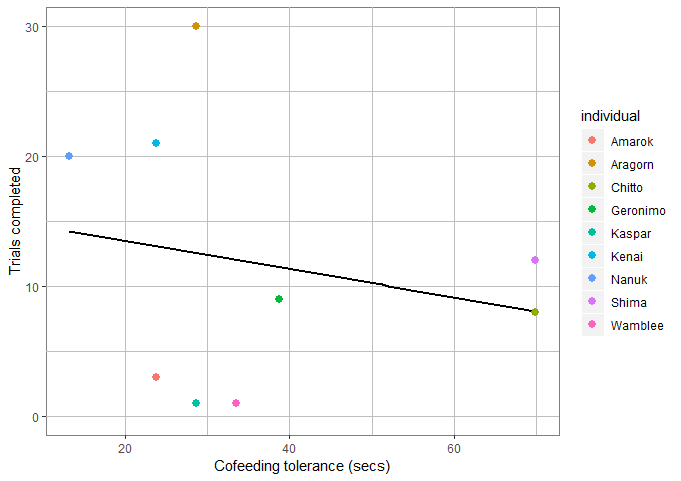


Fig. S6. The higher the feeding tolerance of a dyad, the more inequity averse the subject was.

**
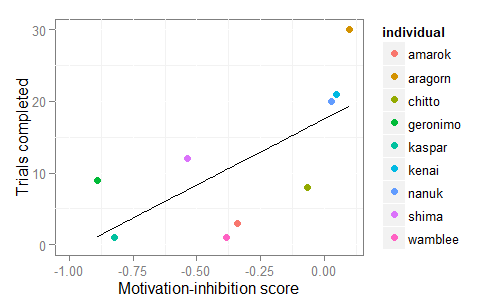
**

Fig. S7. Individuals with a lower motivation score on the inhibition tasks were more inequity averse. A low score represents higher motivation.

Supplementary tables

Table S2. Model comparison for the social influences on cooperation, prosociality and inequity aversion. The model with the highest weight was taken for the final statistics.

| Model components | Df | logLik | AICc | Delta | Weight |
| --- | --- | --- | --- | --- | --- |
| Cooperation | | | | | |
| affiliation + rank distance | 3 | -115-02 | 239.04 | 0.00 | 0.84 |
| affiliation + rank distance + tolerance | 4 | -114.92 | 243.44 | 4.51 | 0.09 |
| rank distance | 2 | -119.80 | 244.93 | 5.89 | 0.04 |
| rank distance + tolerance | 3 | -118.53 | 246.06 | 7.01 | 0.03 |
| affiliation | 2 | -127.59 | 260.51 | 21.47 | 0.00 |
| affiliation + tolerance | 3 | -127.58 | 264.17 | 25.13 | 0.00 |
| tolerance | 2 | -140.39 | 286.11 | 47.07 | 0.00 |
| Null | 1 | -142.76 | 287.91 | 48.87 | 0.00 |
| Prosociality | | | | | |
| affiliation | 2 | -61.77 | 129.54 | 0.00 | 0.74 |
| affiliation + tolerance | 3 | -60.81 | 132.42 | 2.88 | 0.17 |
| affiliation + rank distance | 3 | -61.53 | 133.86 | 4.32 | 0.08 |
| affiliation + rank distance + tolerance | 4 | -60.78 | 139.56 | 10.01 | 0.00 |
| Tolerance | 2 | -73.01 | 152.02 | 22.48 | 0.00 |
| Null | 1 | -76.05 | 154.67 | 25.13 | 0.00 |
| rank distance + tolerance | 3 | -72.80 | 156.41 | 26.87 | 0.00 |
| rank distance | 2 | -75.85 | 157.71 | 28.17 | 0.00 |
| Inequity aversion | | | | | |
| rank distance + tolerance | 3 | -42.21 | 95.21 | 0.00 | 0.63 |
| affiliation + rank distance + tolerance | 4 | -39.32 | 96.64 | 1.43 | 0.31 |
| affiliation + rank distance | 3 | -44.77 | 100.34 | 5.12 | 0.05 |
| rank distance | 2 | -49.19 | 104.38 | 9.17 | 0.01 |
| Tolerance | 2 | -78.58 | 163.16 | 67.95 | 0.00 |
| Null | 1 | -81.29 | 165.15 | 69.93 | 0.00 |
| affiliation + tolerance | 3 | -78.26 | 167.33 | 72.11 | 0.00 |
| affiliation | 2 | -81.29 | 168.57 | 73.36 | 0.00 |

Table S3. Model comparison for the non-social influences on cooperation. The model with the highest weight was taken for the final statistics. Given the large number of models, those with a weight of zero are not included in the table.

| Model components | Df | logLik | AICc | Delta | Weight |
| --- | --- | --- | --- | --- | --- |
| Cooperation | | | | | |
| persistence | 3 | -36.84 | 84.49 | 0.00 | 0.63 |
| Null | 2 | -40.78 | 87.55 | 3.06 | 0.14 |
| Inhibition_perseverance + persistence | 4 | -35.60 | 89.21 | 4.72 | 0.06 |
| Causal understanding + persistence | 4 | -36.40 | 90.80 | 6.31 | 0.03 |
| Inhibition_motivation + persistence | 4 | -36.56 | 91.11 | 6.62 | 0.02 |
| Learning speed + persistence | 4 | -36.59 | 91.17 | 6.68 | 0.02 |
| Inhibition_flexibility + persistence | 4 | -36.78 | 91.56 | 7.07 | 0.02 |
| Learning speed | 3 | -40.48 | 91.76 | 7.27 | 0.02 |
| Causal understanding | 3 | -40.52 | 91.84 | 7.35 | 0.02 |
| Inhibition_perseverance | 3 | -40.59 | 91.97 | 7.48 | 0.02 |
| Inhibition_motivation | 3 | -40.76 | 92.33 | 7.84 | 0.01 |
| Inhibition_flexibility | 3 | -40.78 | 92.35 | 7.86 | 0.01 |
| Prosociality | | | | | |
| Causal understanding + inhibition_motivation + inhibition_perseverance + learning speed + persistence | 7 | -20.33 | -57.33 | 0.00 | 1.00 |
| Null | 2 | -27.82 | 62.65 | 119.98 | 0.00 |
| Inequity aversion | | | | | |
| Causal understanding + inhibition_motivation | 4 | -26.49 | 70.98 | 0.00 | 0.32 |
| inhibition_motivation | 3 | -30.20 | 71.20 | 0.23 | 0.29 |
| Null | 2 | -33.05 | 72.11 | 1.13 | 0.18 |
| Causal understanding | 3 | -32.26 | 75.32 | 4.34 | 0.04 |
| Inhibition_flexibility | 3 | -32.30 | 75.39 | 4.41 | 0.04 |
| Inhibition_flexibility + Inhibition_motivation | 4 | -29.30 | 76.59 | 5.61 | 0.02 |
| Persistence | 3 | -32.90 | 76.59 | 5.61 | 0.02 |
| Learning speed | 3 | -32.91 | 76.62 | 5.64 | 0.02 |
| Inhibition_perseverance | 3 | -33.05 | 76.90 | 5.92 | 0.02 |
| Inhibition_motivation + Inhibition_perseverance | 4 | -29.66 | 77.33 | 6.35 | 0.01 |
| Inhibition_motivation + learning speed | 4 | -29.75 | 77.49 | 6.52 | 0.01 |
| Inhibition_motivation + persistence | 4 | -30.02 | 78.04 | 7.06 | 0.01 |
